# Sepsis Drives Severity and Mortality in Acute-on-Chronic Liver Failure Among ICU Patients with Alcohol-Related Cirrhosis: A Retrospective Single-Center Study

**DOI:** 10.3390/jcm14197025

**Published:** 2025-10-03

**Authors:** Elena von Maldeghem, Katharina Zimmermann, Patricia Mester, Vlad Pavel, Georgios Athanasoulas, Lea Kirsch, David Kolben, Sophia Rusch, Sophie Schlosser-Hupf, Martina Müller, Stephan Schmid

**Affiliations:** Department of Internal Medicine I, Gastroenterology, Hepatology, Endocrinology, Rheumatology, and Infectious Diseases, University Hospital Regensburg, 93053 Regensburg, Germany; elena.maldeghem@ukr.de (E.v.M.); katharina.zimmermann@ukr.de (K.Z.); patricia.mester-pavel@ukr.de (P.M.); vlad.pavel@ukr.de (V.P.); georgios.athanasoulas@ukr.de (G.A.); lea1.kirsch@stud.uni-regensburg.de (L.K.); david.kolben@stud.uni-regensburg.de (D.K.); sophia.rusch@ukr.de (S.R.); sophie.schlosser-hupf@ukr.de (S.S.-H.); martina.mueller-schilling@ukr.de (M.M.)

**Keywords:** acute-on-chronic liver failure, alcohol-related cirrhosis, sepsis, precipitating events, intensive care unit, mortality

## Abstract

**Background/Objectives:** Acute-on-chronic liver failure (ACLF) is a life-threatening complication of cirrhosis, characterized by organ failures and high short-term mortality. Alcohol-related cirrhosis is one of the most frequent underlying etiologies of ACLF in Europe. Infections, particularly those leading to sepsis are recognized triggers; however, their relative contribution, clinical features, and prognostic impact in critically ill patients with alcohol-related cirrhosis remain incompletely defined. This study aimed to systematically identify and characterize precipitating events of ACLF in this population and to compare outcomes between sepsis- and non-sepsis-related cases. **Methods:** We conducted a retrospective cohort study including 188 ICU patients with alcohol-related cirrhosis who were treated for ACLF at a tertiary university medical center. ACLF was defined and graded according to the European Association for the Study of the Liver—Chronic Liver Failure Consortium (EASL-CLIF) criteria, and sepsis was diagnosed according to Sepsis-3 definitions. Clinical data, precipitating events, microbiological evidence, organ support requirements, and in-hospital outcomes were systematically analyzed. **Results:** Sepsis was the most frequent precipitating event, identified in 118 patients (62.8%), while 70 patients (37.2%) developed ACLF due to non-septic triggers such as gastrointestinal bleeding. Patients with sepsis-associated ACLF presented with more advanced disease (ACLF grade 2–3 in 80.5% vs. 57.1%, *p* = 0.004), higher Chronic Liver Failure Consortium—Acute-on-Chronic Liver Failure Score (CLIF-C ACLF) scores (median 55 vs. 50, *p* = 0.04), longer ICU stays (median 11 vs. 4.5 days, *p* < 0.001), and markedly higher in-hospital mortality (60.2% vs. 20.0%, *p* < 0.001) compared to patients without sepsis. Pneumonia (48.3%), urinary infections (17.8%) and spontaneous bacterial peritonitis (16.1%) were the leading infectious foci triggering sepsis. Microbiological evidence was obtained in 82.2% of sepsis cases, with frequent polymicrobial infections and opportunistic pathogens including *Enterococcus faecium* and *Candida albicans*. **Conclusions:** In critically ill patients with alcohol-related cirrhosis, infections leading to sepsis are the predominant precipitating event of ACLF and the strongest determinant of short-term prognosis. Compared with non-sepsis triggers, sepsis-associated ACLF is characterized by more severe disease, greater need for organ support, longer ICU stays, and substantially higher mortality. These findings highlight the urgent need for early recognition, rapid diagnostic strategies, and optimized infection management to improve outcomes in this high-risk population.

## 1. Introduction

Acute-on-chronic liver failure (ACLF) has emerged as a distinct clinical entity that is fundamentally different from acute decompensation of cirrhosis without organ failure. It is characterized by the rapid development of organ dysfunction in patients with underlying chronic liver disease and is associated with extremely high short-term mortality rates, particularly in intensive care unit (ICU) populations [1,2,3,4,5,6,7,8]. The European Association for the Study of the Liver—Chronic Liver Failure (EASL-CLIF) Consortium defined ACLF in the landmark CANONIC (Chronic Liver Failure Acute-on-Chronic Liver Failure in Cirrhosis) study, which provided diagnostic and prognostic criteria and demonstrated the strong impact of the number and type of organ failures on outcome [9]. Since then, ACLF has been recognized as a syndrome of systemic inflammation and multi-organ failure rather than an isolated hepatic deterioration [10].

Alcohol-related cirrhosis represents one of the most frequent underlying conditions in patients who develop ACLF [11,12,13]. Harmful alcohol consumption remains a leading cause of advanced liver disease worldwide and is responsible for a considerable proportion of cirrhosis-related hospitalizations in Europe and beyond [14]. Patients with alcohol-related cirrhosis are particularly vulnerable to acute decompensation and ACLF due to impaired hepatocellular regeneration, immune dysfunction, and a high prevalence of comorbidities such as malnutrition and infections [15,16,17,18,19].

Among the various precipitating events of ACLF, infections—and, in particular, those leading to sepsis—are of paramount importance. Cirrhosis-associated immune dysfunction leads to an increased risk of bacterial translocation, impaired pathogen clearance, and susceptibility to both community-acquired and nosocomial infections [20,21,22,23,24,25]. The Sepsis-3 definition, which conceptualizes sepsis as life-threatening organ dysfunction caused by a dysregulated host response to infection, has refined the diagnostic approach and enabled more precise classification of cirrhotic patients with infections [26,27]. Importantly, sepsis not only acts as a trigger for ACLF but also substantially worsens the clinical course by amplifying systemic inflammation, aggravating multi-organ failure, and increasing short-term mortality [28]. Data from the North American Consortium for the Study of End-Stage Liver Disease (NACSELD) and other cohorts confirm that infection-related ACLF represents a subgroup with particularly poor outcomes [29,30].

Despite this knowledge, there remain important gaps. While large multicenter cohorts have described ACLF epidemiology and prognosis, detailed analyses focusing specifically on alcohol-related cirrhosis, the prevalence and type of sepsis as a precipitating event, the underlying infectious foci, microbiological spectrum, and the direct comparison with non-sepsis-associated ACLF in ICU settings are scarce. Moreover, the role of fungal infections and polymicrobial pathogens has been less systematically studied, despite their increasing clinical importance in critically ill cirrhotic patients [31,32].

The present retrospective study was conducted to address these gaps. We systematically analyzed a well-defined cohort of patients with alcohol-related cirrhosis who developed ACLF during hospitalization in a tertiary care ICU.

The study objectives were to:

(i) describe baseline patient characteristics and comorbidities;
(ii) determine the prevalence and causes of sepsis as a precipitating event of ACLF according to Sepsis-3 criteria;
(iii) assess the microbiological profile of bacterial and fungal pathogens;
(iv) compare clinical course, organ support requirements, and outcomes between patients with sepsis-associated and non-sepsis-associated ACLF.

We aimed to provide a comprehensive characterization of this high-risk patient population and to highlight the prognostic impact of sepsis as a precipitating factor of ACLF in alcohol-associated cirrhosis.

## 2. Materials and Methods

### 2.1. Study Design and Ethical Approval

This retrospective, single-center cohort study was conducted at the Department of Internal Medicine I, University Hospital Regensburg, Germany, a tertiary referral center for hepatology and intensive care medicine. The primary aim was to identify, categorize, and characterize precipitating events of acute-on-chronic liver failure (ACLF) in patients with alcohol-related cirrhosis, with a particular focus on the role of sepsis. The study protocol was reviewed and approved by the institutional ethics committee (reference number 23-3508-104). All procedures adhered to the ethical principles of the Declaration of Helsinki and its subsequent amendments. Patient data were fully anonymized prior to entry into the study database.

### 2.2. Patient Identification and Inclusion Criteria

Patients were identified through a structured query of the hospital’s electronic health record system, covering the period from 1 January 2017 to 31 December 2019. Eligible patients were adults (≥18 years) with a documented diagnosis of liver cirrhosis who developed ACLF during their index hospitalization in the ICU. The diagnosis of cirrhosis was established based on clinical presentation, biochemical parameters, abdominal imaging (ultrasound or CT), endoscopic findings of portal hypertension, and, where available, histological confirmation.

Only patients with alcohol as etiology of cirrhosis were included, determined by clinical history, documentation of chronic alcohol misuse, and exclusion of alternative or concomitant causes (e.g., viral hepatitis, autoimmune hepatitis, cholestatic liver diseases, or metabolic disorders). Patients were excluded if they had mixed or unclear etiologies, prior liver transplantation, advanced hepatocellular carcinoma beyond Milan criteria, or incomplete clinical documentation that precluded ACLF classification. A total of 188 consecutive patients fulfilled all inclusion and exclusion criteria and were included in the final analysis.

### 2.3. Definition and Classification of ACLF

The diagnosis and grading of ACLF were based on the EASL-CLIF Consortium criteria as defined in the CANONIC study. ACLF was defined by either (i) failure of two or more organs, or (ii) failure of a single organ in the presence of renal dysfunction (serum creatinine 1.5–1.9 mg/dL) or hepatic encephalopathy grade I–II [6,9].

Each patient was assigned an ACLF grade (1, 2, or 3) according to the number and type of organ failures. In addition, the CLIF-C ACLF score was calculated to provide an estimate of short-term mortality risk. The time of ACLF onset was defined as the first documentation of organ failure fulfilling these criteria during hospitalization [9].

### 2.4. Definition and Assessment of Sepsis

Sepsis was defined according to the Sepsis-3 consensus definition as life-threatening organ dysfunction caused by a dysregulated host response to infection, operationalized by an increase of ≥2 points in the Sequential Organ Failure Assessment (SOFA) score in the context of a documented or suspected infection. Septic shock was diagnosed in patients requiring vasopressor therapy to maintain a mean arterial pressure ≥65 mmHg in combination with serum lactate >2 mmol/L, despite adequate fluid resuscitation [26,33,34,35].

**Figure 1:**
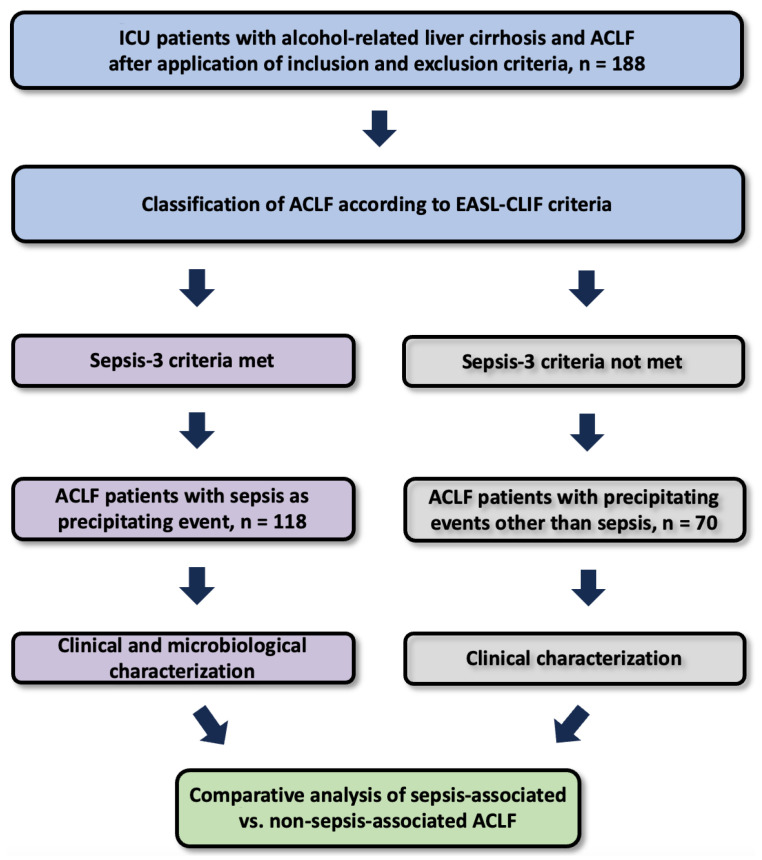
Study Design. A total of 188 ICU patients with alcohol-related cirrhosis and ACLF were classified according to EASL-CLIF criteria and stratified by fulfillment of Sepsis-3 criteria. Patients with sepsis (n = 118) and those without sepsis (n = 70) underwent clinical characterization and were subsequently compared.

For the purposes of this study, patients were stratified into two groups according to the presence or absence of sepsis as precipitating event: (i) sepsis-associated ACLF (n = 118), and (ii) non-sepsis-associated ACLF (n = 70), which served as an internal control group (Figure 1).

### 2.5. Assessment of Precipitating Events

Precipitating events were determined by comprehensive chart review of the period immediately preceding ACLF onset. Data sources included admission notes, hepatology and ICU progress reports, laboratory results, microbiological cultures, radiological imaging, and endoscopy reports. Events were defined as acute clinical insults with a plausible temporal and causal relationship to ACLF onset. In patients with more than one potential precipitant, the event judged to be most temporally associated and clinically relevant was selected as the primary trigger. Classification was independently reviewed by two experienced hepatologists; discrepancies were resolved by consensus.

### 2.6. Microbiological and Infectious Workup

For patients with sepsis-associated ACLF, all available microbiological test results were collected, including blood, urine, ascitic fluid, and respiratory cultures, as well as samples from invasive procedures such as bronchoscopy. Pathogens were categorized as Gram-positive, Gram-negative, or fungal, and cases with detection of multiple organisms were classified as polymicrobial infections. The anatomical source of infection (e.g., pneumonia, spontaneous bacterial peritonitis, urinary tract infection) was recorded according to clinical and microbiological findings.

### 2.7. Data Collection and Variables

A standardized data collection form was used to extract patient demographics (age, sex, body mass index), comorbidities, history of decompensation, and etiology of cirrhosis. Laboratory parameters included liver biochemistry, coagulation profile, renal function, inflammatory markers (e.g., C-reactive protein, procalcitonin), and arterial blood gas analysis. Clinical scores (MELD, CLIF-C OF, CLIF-C ACLF, SOFA) were calculated at admission.

Therapeutic interventions and organ support measures (renal replacement therapy, vasopressor requirement, mechanical or non-invasive ventilation) were documented, as were ICU and hospital length of stay. Outcomes included in-hospital mortality, discharge, transfer to other facilities, and discharge against medical advice.

### 2.8. Statistical Analysis

Statistical analyses were performed using IBM SPSS Statistics (Version 28.0.0.0, IBM Corp., Armonk, NY, USA). Continuous variables were summarized as mean ± standard deviation (SD) or median with interquartile range (IQR), depending on distribution. Categorical variables were expressed as counts and percentages.

Comparisons between groups (e.g., sepsis-associated vs. non-sepsis-associated ACLF) were performed using the Mann–Whitney U test or Student’s *t*-test for continuous variables and chi-square or Fisher’s exact test for categorical variables, as appropriate. A two-sided *p*-value <0.05 was considered statistically significant. Missing values were not imputed, and analyses were conducted based on available data.

Multivariate logistic regression analysis was performed to identify independent predictors of in-hospital mortality. Variables with a *p*-value <0.05 in univariate analyses, as well as variables considered clinically relevant, were included in the model. Results are reported as adjusted odds ratios (OR) with corresponding 95% confidence intervals (CI).

## 3. Results

### 3.1. Patient Population and Baseline Characteristics

A total of 188 patients with alcohol-related cirrhosis who were hospitalized on the ICU due to acute-on-chronic liver failure (ACLF) were identified and included in this retrospective analysis.

All patients met the diagnostic criteria for ACLF as defined by the EASL-CLIF Consortium. The underlying etiology of liver disease was confirmed as alcohol-related based on review of medical records, clinical assessment, and exclusion of alternative causes of chronic liver disease.

The median age of the study cohort was 57 years (interquartile range [IQR] 50.3–62.8). Of these patients, 45 (23.9%) were female and 143 (76.1%) were male. The mean BMI was 25.7 (interquartile range [IQR] 23.6–29.4). The median Model for End-Stage Liver Disease (MELD) score at the time of hospital admission was 34 (IQR 28.0–38.5). According to the Child–Pugh classification, the majority of patients presented with advanced disease: 84.0% were categorized as Child–Pugh class C, 13.8% as class B, and 2.1% as class A. With respect to ACLF severity, 53 patients (28.2%) were diagnosed with ACLF grade 1, 74 patients (38.4%) with ACLF grade 2, and 61 patients (32.4%) with ACLF grade 3. The mean CLIF-C ACLF score was 53 (IQR 45–60), reflecting a cohort with a high short-term mortality risk.

Relevant comorbidities included type 2 diabetes mellitus in 22.3% of patients. A pre-existing malignant disease was documented in 14.9% of cases, with hepatocellular carcinoma (HCC) being the most prevalent neoplastic condition.

According to the admission notes, the leading reasons for hospital presentation were deterioration of general condition (23.4%), hepatic encephalopathy (19.7%), and pain (11.2%). Less frequent reasons for admission included respiratory insufficiency (5.3%), diarrhea (4.8%), and hyperglycemia (4.3%). These findings illustrate the broad clinical spectrum with which patients with decompensated alcohol-related cirrhosis and ACLF are presented to the hospital, ranging from nonspecific decline to acute complications directly attributable to liver failure and its systemic consequences.

**Figure 2:**
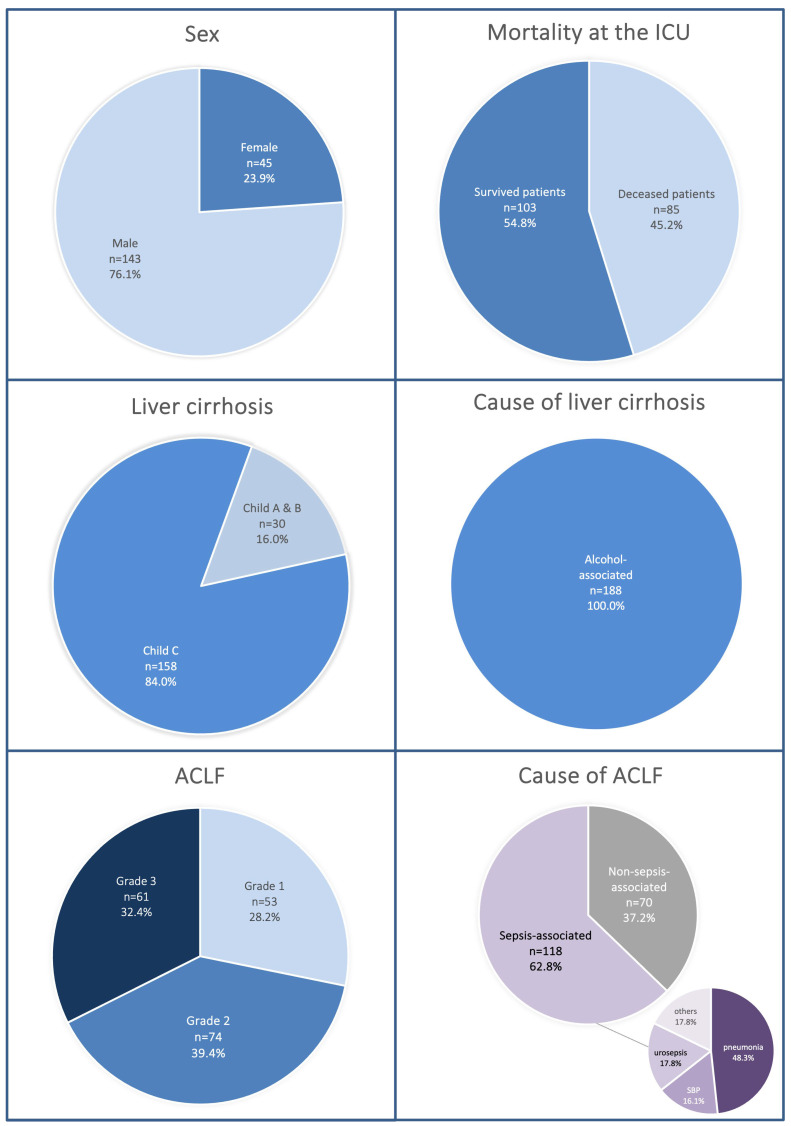
Baseline characteristics and precipitating events of the study cohort (n = 188). Most patients were male (76.1%), classified as Child C (84.0%), and presented with advanced ACLF (grades 2–3: 71.8%). Overall ICU mortality was 45.2%. Sepsis was the leading precipitating event (62.8%), most commonly due to pneumonia, urosepsis, or spontaneous bacterial peritonitis.

The median duration of ICU stay in the overall cohort was 7 days (IQR 3–16). In terms of outcome, 85 patients (45.2%) died during the index hospitalization, 74 patients (39.9%) were discharged home after subsequent transfer to a general ward within the same hospital, 14 patients (7.4%) were transferred to another hospital, 9 patients (4.8%) left the hospital against medical advice, and 6 patients (3.2%) were transferred to a specialized neurorehabilitation facility. These findings underscore the high overall mortality and the heterogeneous clinical trajectories of patients with alcohol-related cirrhosis and ACLF admitted to the ICU (Table 1, Figure 2).

### 3.2. Sepsis as a Precipitating Event of ACLF in Patients with Alcohol-Related Cirrhosis

Among the 188 patients with alcohol-related cirrhosis who were hospitalized due to ACLF, sepsis was identified as the precipitating event in 118 patients (62.8%).

The median age in this subgroup was 57.8 years (interquartile range [IQR] 50.8–63.3). A total of 29 patients (24.9%) were female and 89 patients (75.4%) male. The mean BMI was 26.6 (IQR 23.3–29.8). With respect to liver disease severity, the vast majority of patients were classified as Child–Pugh class C (83.9%, n = 99), while 14.4% (n = 17) were Child–Pugh class B and 1.7% (n = 2) Child–Pugh class A. The median Child–Pugh score was 12 (IQR 10–13). The median MELD score on admission was 33 (IQR 27.0–37.3), reflecting severely advanced liver disease.

According to the EASL-CLIF ACLF classification, most patients presented with ACLF grade 2 (44.1%, n = 52), followed by grade 3 (36.4%, n = 43), and grade 1 (19.5%, n = 23). The CLIF-C ACLF score was ≥50 in the majority of cases (65.3%, n = 77), while 15.3% (n = 18) had values between 40 and 50 and 19.5% (n = 23) had values <40. The overall median CLIF-C ACLF score in this subgroup was 55 (IQR 47.5–61), consistent with a high short-term mortality risk.

The median length of stay in the intensive care unit was 11 days (IQR 5–22). Outcome analysis revealed that 60.2% (n = 71) of patients with sepsis-associated ACLF died during the hospitalization. In contrast, 22.9% (n = 27) were discharged home after subsequent transfer to a general ward within the same hospital, 7.6% (n = 9) were transferred to other hospitals, 5.9% (n = 7) left the hospital against medical advice, and only 3.4% (n = 4) could be transferred to a rehabilitation facility.

These findings underscore that in patients with alcohol-related cirrhosis, sepsis is a frequent precipitating event for ACLF and is associated with particularly advanced liver disease at baseline, high organ failure burden, and poor clinical outcomes.

### 3.3. Etiology and Clinical Features of Sepsis in Patients with ACLF

In patients with sepsis-associated ACLF (n = 118), pneumonia was the most frequently identified infectious focus, diagnosed in 57 cases (48.3%). Among these, 25.4% (30 cases) were classified as pneumonia without further specification, 15.3% (18 cases) as aspiration pneumonia, 5.1% (6 cases) as *Pneumocystis jirovecii* pneumonia, and 2.5% (3 cases) as nosocomial pneumonia.

Urosepsis and spontaneous bacterial peritonitis emerged as the most frequent non-pulmonary infectious foci in this cohort, identified in 21 patients (17.8%) and 19 patients (16.1%), respectively. Cholangiosepsis was documented in four patients (3.4%). Less common foci, observed in two patients each (1.7%), included urinary tract infections, intra-abdominal abscesses, peritonitis not fulfilling the criteria of SBP, and bloodstream infections. Rare causes, seen in a single patient each (0.8%), were wound infection, abdominal sepsis of unclear origin, and soft tissue infection. In addition, in eight patients (6.8%) no definite septic focus could be established despite extensive diagnostic evaluation.

The high prevalence of SBP and urosepsis underlines their particular relevance as frequent and equally common precipitants of sepsis-associated ACLF in patients with alcohol-related cirrhosis, reflecting the central role of bacterial translocation and urinary tract vulnerability in this critically ill population.

The median Sequential Organ Failure Assessment (SOFA) score at sepsis diagnosis was 10.5 (IQR 8–14), reflecting a critically ill population with a high burden of organ dysfunction. Septic shock, according to Sepsis-3 definitions, was present in 29.7% of cases.

The mean CRP level on admission was 25.1 mg/L (IQR 12.7–49.4), and the mean procalcitonin (PCT) level was 0.58 ng/mL (IQR 0.31–1.38). The mean leukocyte count was 13.3/nL (IQR 8.7–21.9). Liver enzymes were elevated, with a mean ALT of 41 U/L (IQR 18–65.4) and a mean AST of 83 U/L (IQR 42–170). Bilirubin levels averaged 9.2 mg/dL (IQR 3.2–17.4), and the mean gamma-glutamyl transferase (GGT) was 70.4 U/L (IQR 35.8–150.3). The mean Quick value was 37% (IQR 28–48), and the mean INR was 1.9 (IQR 1.6–2.4). Platelet counts averaged 68/nL (IQR 42.5–119.5), while the mean albumin concentration was 23.5 g/L (IQR 19.1–29.4).

With regard to organ support requirements, 52.2% (n = 62) of patients required vasopressor therapy, 42.4% (n = 50) required invasive or non-invasive respiratory support, and 15.3% (n = 18) underwent renal replacement therapy during the course of sepsis management.

These findings emphasize that in patients with alcohol-related cirrhosis and ACLF, sepsis is most commonly triggered by pneumonia, SBP or urosepsis, frequently requires advanced organ support, and is associated with high rates of septic shock and multi-organ failure (Table 2).

### 3.4. Microbiological Evidence in Patients with Sepsis-Associated ACLF

In the subgroup of patients with sepsis-associated ACLF and underlying alcohol-related cirrhosis (n = 118), microbiological evidence of infection was obtained in 97 patients (82.2%). Among these, Gram-positive bacteria were isolated in 66.0% (n = 64), while Gram-negative bacteria were identified in 32.0% (n = 31). In addition, fungal pathogens were detected in a relevant proportion of cases.

Polymicrobial infections were common: three distinct pathogens were identified in 29.9% (n = 29) of patients with positive microbiological findings, two pathogens in 18.6% (n = 18), and a single pathogen in 21.9% (n = 21).

The most frequently isolated organisms included *Enterococcus faecium* (17.5%, n = 17), *Candida albicans* (13.4%, n = 13), and *Escherichia coli* (7.2%, n = 7). Other relevant pathogens were *Klebsiella pneumoniae* (6.2%, n = 6), *Enterobacter aerogenes* (6.2%, n = 6), *Staphylococcus epidermidis* (4.1%, n = 4), and *Enterobacter cloacae* (4.1%, n = 4). Pathogens were most frequently isolated from bronchial secretions (11.3%, n = 11), followed by blood cultures (8.2%, n = 8). In 7.2% of cases, pathogen detection originated from urine cultures (n = 7) or bronchoscopy samples (n = 7).

These findings illustrate the high prevalence of microbiological confirmation in sepsis-associated ACLF, the frequent presence of polymicrobial infections, and the clinical relevance of both bacterial and fungal pathogens in this patient population (Table 3).

### 3.5. ACLF Precipitating Events Other than Sepsis in Patients with Alcohol-Related Cirrhosis (=Control Group)

Beyond sepsis, other clinical conditions such as hepatic encephalopathy and gastrointestinal bleeding were identified as precipitating events of ACLF in patients with alcohol-related cirrhosis. In this dataset, 70 of the 188 patients (37.2%) presented with ACLF in the absence of sepsis.

The median age in this subgroup was 55 years (IQR 50.0–61.3). Female patients accounted for 22.9% (n = 16), while 77.1% (n = 54) were male. The mean BMI was 25.0 (IQR 23.6–29.2). The majority of patients were classified as Child–Pugh class C (84.3%, n = 59), whereas 12.9% (n = 9) were Child–Pugh class B and 2.9% (n = 2) Child–Pugh class A. The median Child–Pugh score was 11 (IQR 10–13), and the median MELD score was 30 (IQR 24.0–37.3).

With respect to ACLF severity according to the EASL-CLIF criteria, most patients presented with ACLF grade 1 (42.9%, n = 30), followed by grade 2 (31.4%, n = 22) and grade 3 (25.7%, n = 18). The median CLIF-C ACLF score was 50 (IQR 43–57).

The median ICU length of stay in this subgroup was 4.5 days (IQR 2.0–7.3), which was markedly shorter compared to patients with sepsis-associated ACLF. In-hospital mortality was 20.0% (n = 14), while 67.1% (n = 47) of patients were discharged. A further 7.1% (n = 5) were transferred to other hospitals, 2.9% (n = 2) were discharged against medical advice, and 2.9% (n = 2) were transferred to rehabilitation facilities.

Inflammatory biomarkers in this group were as follows: The mean CRP at admission was 22.8 mg/L (IQR 9.5–47.6), the mean PCT 0.52 ng/mL (IQR 0.19–1.28), and the leukocyte count 8.7/nL (IQR 5.4–13.2). Liver enzymes were moderately elevated, with a mean ALT of 33.4 U/L (IQR 19.4–52.3) and a mean AST of 62 U/L (IQR 38.4–117.8). Bilirubin showed a mean of 6.0 mg/dL (IQR 2.4–24.0), while gamma-glutamyl transferase averaged 70 U/L (IQR 35.0–143.4). The mean Quick value was 41% (IQR 30–47.3), with a mean INR of 1.7 (IQR 1.6–2.3). Platelet counts averaged 63.4/nL (IQR 46.8–98.3), and mean serum albumin was 25.9 g/L (IQR 20.7–32.0).

Regarding organ support, 35.7% (n = 25) required vasopressor therapy, 25.7% (n = 18) required respiratory support, and 2.9% (n = 2) underwent renal replacement therapy.

Taken together, these findings demonstrate that patients with non-sepsis-associated ACLF presented with lower ACLF grades, shorter ICU stays, and markedly lower mortality compared to those with sepsis-associated ACLF, as summarized in Table 4.

### 3.6. Comparison Between Patients with Sepsis-Associated and Non-Sepsis-Associated ACLF

**Figure 3:**
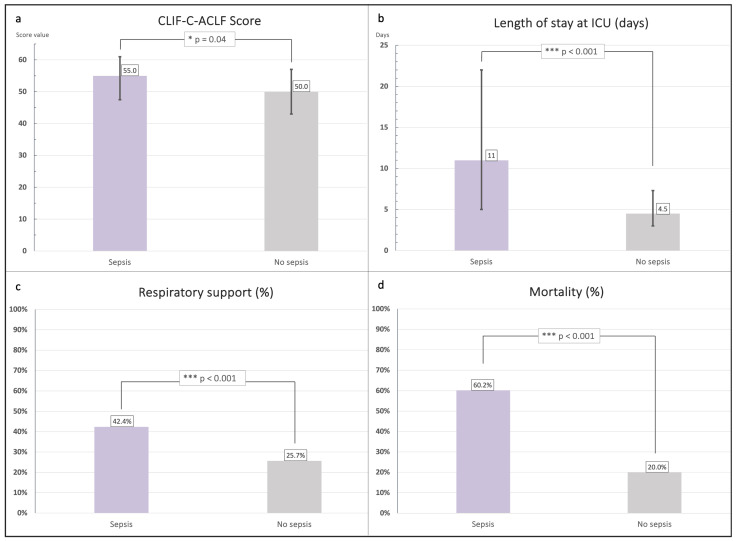
Clinical outcomes in patients with sepsis- and non-sepsis-associated ACLF. Compared to non-sepsis-associated cases, patients with sepsis-associated ACLF showed significantly higher CLIF-C ACLF scores (median 55 vs. 50, *p* = 0.04; panel (**a**)), longer ICU stays (median 11 vs. 4.5 days, *p* < 0.001; panel (**b**)), more frequent need for respiratory support (42.4% vs. 25.7%, *p* < 0.001; panel (**c**)), and markedly higher in-hospital mortality (60.2% vs. 20.0%, *p* < 0.001; panel (**d**)). Asterisks indicate the level of statistical significance: *p* < 0.05 (*), *p* < 0.001 (***).

When comparing patients with sepsis-associated ACLF (n = 118) to those with non-sepsis-associated ACLF (n = 70), several relevant differences were observed (Figure 3). Patients in the sepsis group were slightly older (median age 57.8 years, IQR 50.8–63.3) compared to the non-sepsis group (median age 55.0 years, IQR 50.0–61.3), although this difference did not reach statistical significance (*p* = 0.43).

Baseline liver disease severity was similar between the two groups. The distribution of Child–Pugh classes (C: 83.9% vs. 84.3%) and median Child–Pugh score (12 [IQR 10–13] vs. 11 [IQR 10–13]) showed no significant difference (*p* = 0.37). Median MELD scores were slightly higher in the sepsis group (33 [IQR 27.0–37.3]) compared to the non-sepsis group (30 [IQR 24.0–37.3]), but again without statistical significance (*p* = 0.19).

In contrast, significant differences were noted with respect to ACLF severity. Patients with sepsis more frequently presented with advanced ACLF (grade 2 or 3: 80.5% vs. 57.1%), whereas patients without sepsis more often exhibited ACLF grade 1 (42.9% vs. 19.5%; *p* = 0.004). This was reflected in higher CLIF-C ACLF scores in the sepsis group (median 55, IQR 47.5–61) compared to the non-sepsis group (median 50, IQR 43–57; *p* = 0.04).

The median ICU length of stay was substantially longer among patients with sepsis (11 days, IQR 5–22) compared with those without sepsis (4.5 days, IQR 2.0–7.3; *p* < 0.001). Mortality was also markedly higher in the sepsis group (60.2%) than in the non-sepsis group (20.0%; *p* < 0.001).

Organ support requirements further underlined the differences between groups. The need for vasopressor therapy was significantly more frequent in sepsis-associated ACLF (52.2% vs. 35.7%; *p* = 0.02). Similarly, the proportion of patients requiring renal replacement therapy was higher in the sepsis group (15.3% vs. 2.9%; *p* < 0.001). Respiratory support was required in 42.4% of sepsis patients compared to 25.7% of non-sepsis patients (*p* < 0.001).

These results demonstrate that sepsis is not only the most frequent precipitating event but also the strongest determinant of disease severity and outcome in patients with alcohol-related cirrhosis and ACLF. Compared with ACLF precipitated by non-infectious events, patients with sepsis presented with significantly higher ACLF grades and CLIF-C ACLF scores, reflecting a more advanced stage of systemic failure. They required organ support more frequently, including vasopressor therapy, early respiratory support, and renal replacement therapy, and remained in intensive care for a substantially longer period.

Most importantly, in-hospital mortality was almost three times higher in sepsis-associated ACLF compared to non-sepsis-associated cases. These findings underscore the important role of sepsis as both a trigger and an amplifier of ACLF progression and provide the rationale for the subsequent discussion on pathophysiological mechanisms, clinical implications, and future research directions.

In the multivariable logistic regression analysis adjusting for age, sex, MELD score, and ACLF grade, sepsis emerged as the strongest independent predictor of in-hospital mortality (Table 5). Patients with sepsis-associated ACLF had a nearly fivefold higher risk of death compared to those without sepsis (OR 4.68, 95% CI 2.31–9.51, *p* < 0.001). In addition, a higher ACLF grade (grade 2–3 vs. grade 1) was independently associated with an approximately twofold increased risk of in-hospital mortality (adjusted OR 2.07, 95% CI 1.28–3.37, *p* = 0.003). By contrast, age, sex, and MELD score were not significantly associated with mortality in this model.

**Figure 4:**
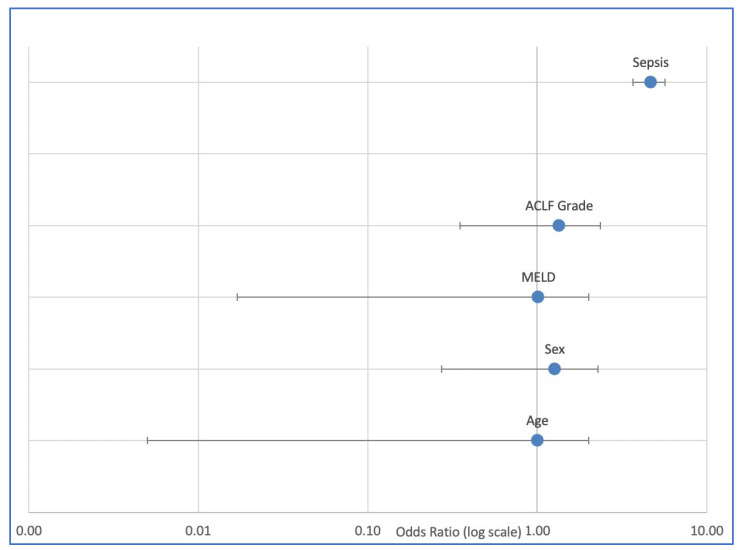
Multivariable logistic regression for in-hospital mortality in patients with alcohol-related cirrhosis and ACLF (n = 188). Sepsis (OR 4.68, 95% CI 2.31–9.51, *p* < 0.001) and higher ACLF grade (OR 2.07, 95% CI 1.28–3.37, *p* = 0.003) were independently associated with increased risk of death, while age, sex, and MELD score were not significant predictors.

These findings confirm that sepsis represents not only the most frequent precipitating event of ACLF but also the most relevant determinant of short-term outcome in critically ill patients with alcohol-related cirrhosis. The prognostic relevance of ACLF severity further underscores the importance of early recognition of multi-organ failure (Figure 4).

When including the SOFA score in the multivariable model (Table 6), sepsis remained the strongest independent predictor of in-hospital mortality (adjusted OR 4.88, 95% CI 2.37–10.03, *p* < 0.001). In addition, the SOFA score itself was significantly associated with mortality, with each one-point increase corresponding to a 15% higher risk of death (adjusted OR 1.15, 95% CI 1.02–1.28, *p* = 0.019). By contrast, ACLF grade, age, sex, and MELD score did not show significant independent associations with outcome in this adjusted model.

**Figure 5:**
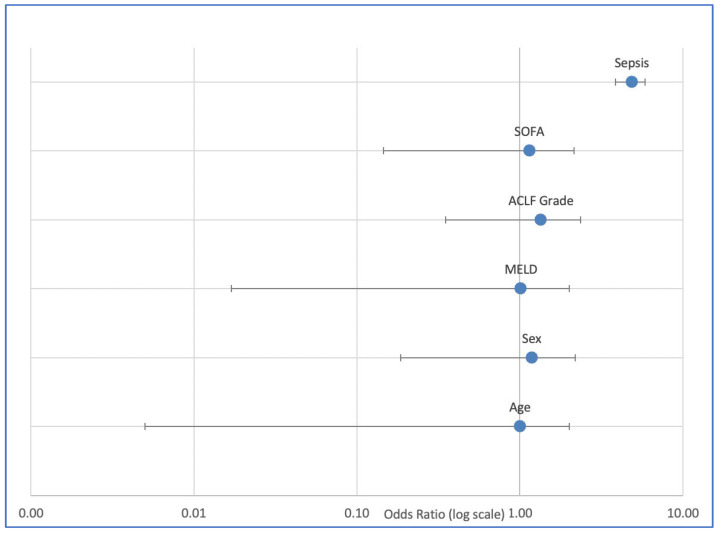
Multivariable logistic regression including SOFA score for in-hospital mortality in patients with alcohol-related cirrhosis and ACLF (n = 188). Sepsis (OR 4.88, 95% CI 2.37–10.03, *p* < 0.001) and SOFA score (OR 1.15 per point, 95% CI 1.02–1.28, *p* = 0.019) were independently associated with mortality, while age, sex, MELD score, and ACLF grade did not retain significance.

These findings demonstrate that, while both ACLF grade and SOFA are markers of organ dysfunction, the SOFA score better captures the prognostic impact of acute organ failures in this critically ill cohort. Importantly, sepsis retained its role as the predominant determinant of mortality even after adjusting for disease severity (Figure 5).

## 4. Discussion

In this retrospective cohort of 188 critically ill patients with alcohol-related cirrhosis who developed acute-on-chronic liver failure (ACLF), we found that sepsis—as defined by the Sepsis-3 consensus criteria—was by far the most frequent precipitating event, occurring in nearly two-thirds of cases. Non-septic precipitants, including gastrointestinal bleeding, hepatic encephalopathy, or multifactorial decompensation, accounted for the remaining one-third. Importantly, sepsis not only emerged as the predominant trigger, but also as the most deleterious, with significantly higher ACLF grades, greater organ support requirements, prolonged ICU stay, and markedly increased short-term mortality compared to non-sepsis-associated ACLF.

Our findings extend those of the CANONIC study, which established bacterial infection as the most common precipitant of ACLF, and are consistent with data from the NACSELD consortium and other large registries [3,29,36,37,38,39,40]. However, the proportion of sepsis-associated ACLF in our ICU cohort (62.8%) was even higher than that reported in multicenter studies, where prevalence rates ranged between 30 and 50% [41]. This difference likely reflects differences in patient selection: while registries also include patients with less severe disease on general wards, our cohort exclusively comprised ICU patients with advanced alcohol-associated cirrhosis and high MELD and Child–Pugh scores, thus representing a population at exceptional risk for infection-related complications.

The pathophysiological link between alcohol-associated cirrhosis, infection, and ACLF is multifaceted. Chronic alcohol consumption impairs both innate and adaptive immunity through mechanisms such as defective neutrophil chemotaxis, reduced phagocytic capacity, altered macrophage function, and diminished complement activity [42]. In parallel, alcohol-associated cirrhosis promotes gut dysbiosis and increased intestinal permeability, thereby facilitating microbial translocation and persistent systemic inflammation [43,44]. Once infection occurs, these patients mount a dysregulated host response, characterized by excessive pro-inflammatory cytokine release and immunoparalysis, which accelerates organ failure. This mechanism is in line with the concept of systemic inflammation as the pathophysiological hallmark of ACLF proposed by the EASL-CLIF Consortium [45].

The clinical consequences in our cohort were profound. Patients with sepsis-associated ACLF presented with higher CLIF-C ACLF scores (median 55 vs. 50) and more frequently exhibited ACLF grade 2 or 3. They required more intensive organ support: over half needed vasopressors, more than 40% required respiratory support within the first five hours of ICU admission, and 15% underwent renal replacement therapy. This translated into a hospital mortality of 60%, three times higher than in patients with non-sepsis-associated ACLF (20%). These results clearly illustrate that sepsis is not merely a common trigger but a determinant of prognosis in alcohol-relatedACLF.

In addition to established inflammatory biomarkers such as C-reactive protein and procalcitonin, novel hepatic enzymes have recently gained attention as potential prognostic markers in critically ill patients. Among these, serum butyrylcholinesterase (BChE) has been reported to correlate with systemic inflammation, infection severity, and outcomes in ICU cohorts. Low BChE activity has been associated with the development of septic complications, particularly in postoperative patients, and may thus reflect both impaired hepatic synthetic capacity and the systemic inflammatory burden. [46,47,48]. Although BChE levels were not assessed in our study, their potential role as an early and easily measurable predictor of sepsis and poor outcome in cirrhotic ICU patients is of considerable interest. Future prospective studies should further evaluate the diagnostic and prognostic value of BChE in comparison with established inflammatory biomarkers to refine risk stratification in this vulnerable population.

Our detailed analysis of infectious foci further highlights important clinical implications. Pneumonia accounted for nearly half of all sepsis cases, consistent with previous ICU-based series [49]. Aspiration pneumonia was frequent, reflecting the vulnerability of cirrhotic patients with encephalopathy, impaired airway protection, and high prevalence of alcohol-related malnutrition and sarcopenia. Spontaneous bacterial peritonitis (SBP) was the second most common focus, underlining its continued importance despite routine prophylaxis in many cirrhotic patients. Strikingly, in 12.7% of cases no infectious focus could be identified, a finding echoed in other studies, which may point to under-detection of subtle infections [50].

Microbiological analyses revealed high rates of pathogen detection (82%), frequent polymicrobial infections, and a substantial contribution of opportunistic organisms. Enterococcus faecium was the most frequently isolated bacterium, followed by Candida albicans and Escherichia coli. The prominence of enterococci and Candida is of particular concern, as these pathogens are often associated with nosocomial acquisition, multidrug resistance, and poor outcomes [51]. Fungal infections, present in over 10% of our cohort, represent a growing challenge in cirrhosis, yet remain underappreciated in many epidemiological studies [52]. These results underscore the need for heightened awareness, early diagnostic strategies, and consideration of fungal coverage in selected high-risk ICU patients.

By contrast, patients with non-septic ACLF displayed a distinctly different clinical course. Although they had similarly advanced liver disease at baseline (median MELD 30, 84% Child–Pugh C), they more often presented with ACLF grade 1 and required less organ support. Their ICU stay was significantly shorter (median 4.5 vs. 11 days), and in-hospital mortality was much lower (20%). Gastrointestinal bleeding was a frequent precipitant in this group. Advances in endoscopic and pharmacologic therapy, alongside early antibiotic prophylaxis, may explain the relatively favorable outcomes compared with earlier eras. Hepatic encephalopathy as a precipitant reflects metabolic decompensation rather than systemic inflammation and is therefore less frequently associated with multi-organ failure.

Taken together, our results provide compelling evidence that the type of precipitating event strongly influences the trajectory and outcome of ACLF in patients with alcohol-related cirrhosis. Sepsis emerges as the most devastating precipitant, leading to higher disease severity, prolonged ICU stays, and excessive mortality. This highlights the urgent need for early recognition of infection in cirrhotic patients, aggressive empiric anti-infective treatment, and rigorous infection control measures in the ICU. Furthermore, the predominance of nosocomial and opportunistic pathogens such as enterococci and Candida calls for a re-evaluation of empirical treatment strategies, particularly in high-risk ICU populations.

Our study has several strengths, including the relatively large and etiologically homogeneous cohort, systematic classification of ACLF according to EASL-CLIF criteria, and rigorous application of Sepsis-3 definitions. The detailed microbiological analysis provides additional granularity that is lacking in many registry studies. However, limitations must also be acknowledged. The retrospective, single-center design introduces risks of selection bias and limits generalizability

Despite these limitations, our findings underscore the central role of sepsis as the predominant and prognostically most relevant precipitant of ACLF in alcohol-related cirrhosis. Importantly, with 188 ICU patients included, this study represents one of the largest cohorts to date focusing specifically on alcohol-related cirrhosis complicated by ACLF, thereby providing robust evidence that strengthens the clinical relevance of our observations. Clinically, these results support a strategy of heightened vigilance, rapid sepsis work-up, and aggressive infection management in all cirrhotic patients admitted with ACLF. Future prospective studies should further delineate pathogen-specific outcomes, evaluate the role of biomarkers and rapid molecular diagnostics for early infection detection, and explore preventive strategies—including selective intestinal decontamination, antifungal prophylaxis in high-risk groups, and improved vaccination coverage—to mitigate the burden of sepsis-related ACLF in this highly vulnerable population.

## 5. Conclusions

In this retrospective analysis of 188 ICU patients with alcohol-related cirrhosis and ACLF, sepsis—as defined by Sepsis-3 criteria—was identified as the precipitating event in nearly two-thirds of cases. Compared with non-sepsis-associated ACLF, sepsis-triggered ACLF was characterized by higher CLIF-C ACLF scores, more advanced disease grades, greater need for vasopressor, respiratory, and renal replacement therapy, prolonged ICU stays, and markedly increased in-hospital mortality (60% vs. 20%). Pneumonia was the most frequent infectious focus, followed by spontaneous bacterial peritonitis, while microbiological evidence revealed a high prevalence of polymicrobial infections and opportunistic pathogens, notably Enterococcus faecium and Candida albicans.

These findings highlight that sepsis is not only the most common precipitant but also the major driver of severity and prognosis in ACLF in patients with alcohol-related liver cirrhosis. From a clinical perspective, this underscores the critical importance of systematic sepsis screening, rapid initiation of empirical anti-infective treatment, and rigorous infection control strategies in cirrhotic patients admitted with ACLF. To sum up, early recognition and targeted management of sepsis are essential to mitigate progression, improve outcomes, and ultimately reduce mortality in this vulnerable population.

## Figures and Tables

**Table 1:** Clinical characteristics of the total study population (n = 188).

Characteristics	Total Study Population (n = 188)
Age, years, mean [IQR]	57 [50.25–62.75]
Sex, n (%)	
Female	45 (23.9)
Male	143 (76.1)
BMI, median [IQR]	25.7 [23.6–29.4]
Child–Pugh classification, n (%)	
Child–Pugh-Class A	4 (2.1)
Child–Pugh-Class B	26 (13.8)
Child–Pugh-Class C	158 (84.0)
MELD score, median [IQR]	34 [28–38.5]
ACLF grade, n (%)	
Grade 1	53 (28.2)
Grade 2	74 (38.4)
Grade 3	61 (32.4)
CLIF-C-ACLF score, median [IQR]	53 [45–60]
Duration of ICU stay, days, median [IQR]	7 [3–16]
Outcome, n (%)	
Death	85 (45.2)
Discharged after subsequent transfer to general ward	74 (39.9)
Transfer to another ICU	14 (7.4)
Discharge against medical advice	9 (4.8)
Transfer to neurorehabilitation unit	6 (3.2)
Leading Symptoms at admission, n (%)	
Deterioration of general condition	44 (23.4)
Hepatic encephalopathy	37 (19.7)
Pain	21 (11.2)
Respiratory insufficiency	10 (5.3)
Diarrhea	9 (4.8)
Hyperglycemia	8 (4.3)

Baseline characteristics, disease severity scores, clinical presentation at hospital admission, and in-hospital outcomes of all patients with alcohol-related cirrhosis who developed acute-on-chronic liver failure (ACLF) during the index hospitalization. Values are presented as numbers (percentages) or median with interquartile range (IQR), as appropriate.

**Table 2:** Clinical characteristics of the patients with sepsis-associated ACLF (n = 118).

Characteristics	Study Population (n = 118)
Age, years, median [IQR]	57.75 [50.75–63.25]
Sex, n (%)	
Female	29 (24.9)
Male	89 (75.4)
BMI, median [IQR]	26.6 [23.3–29.8]
Child–Pugh classification, n (%)	
Child–Pugh Class A	2 (1.7)
Child–Pugh Class B	17 (14.4)
Child–Pugh Class C	99 (83.9)
MELD score, median [IQR]	33 [27–37.25]
ACLF grade, n (%)	
Grade 1	23 (19.5)
Grade 2	52 (44.1)
Grade 3	43 (36.4)
CLIF-C-ACLF score, median [IQR]	55 [47.5–61]
Duration of ICU stay, days, median [IQR]	11 [5–22]
Outcome, n (%)	
Death	71 (60.2)
Discharged after subsequent transfer to general ward	27 (22.9)
Transfer to another ICU	9 (7.6)
Discharge against medical advice	7 (5.9)
Transfer to neurorehabilitation unit	4 (3.4)
Infectious foci, n (%)	
Pneumonia	57 (48.3)
Urosepsis	21 (17.8)
Spontaneous bacterial peritonitis (SBP)	19 (16.1)
Cholangiosepsis	4 (3.4)
Intra-abdominal abscess	2 (1.7)
Peritonitis	2 (1.7)
Bloodstream infection	2 (1.7)
Wound infection	1 (0.8)
Abdominal sepsis (unclear origin)	1 (0.8)
Soft tissue infection	1 (0.8)
No focus identified	8 (6.8)
SOFA score, median [IQR]	10.5 [8–14]
Laboratory parameters, median [IQR]	
CRP, mg/L	25.1 [12.7–49.4]
PCT, ng/mL	0.58 [0.31–1.38]
Leukocytes, /nL	13.3 [8.7–21.9]
ALT, U/L	41 [18–65.4]
AST, U/L	83 [42–170]
gGT, U/L	70.4 [35.8–150.3]
Bilirubin mg/dL	9.2 [3.2–17.4]
Quick, %	37 [28–48]
INR	1.9 [1.6–2.4]
Thrombocytes, /nL	68 [42.5–119.5]
Albumin, g/L	23.5 [19.1–29.4]
Organ support, n (%)	
Circulatory support (vasopressor therapy)	62 (52.2)
Respiratory support within 5 h of admission	50 (42.4)
Renal replacement therapy	18 (15.3)

Demographics, disease severity, outcomes, laboratory parameters, and infectious foci in patients with alcohol-related cirrhosis and sepsis-associated ACLF. Values are given as numbers (percentages) or median with interquartile range (IQR). Pneumonia was the most frequent infectious focus, followed by urosepsis and spontaneous bacterial peritonitis.

**Table 3:** Clinical characteristics of the patients with sepsis-associated ACLF and microbiological evidence (n = 97).

Characteristics	Study Population (n = 97)
Bacteria	
Gram-positive	64 (66.0)
Gram-negative	31 (32.0)
Culture medium, n (%)	
Bronchial secretions	11 (11.3)
Blood cultures	8 (8.2%)
Urine cultures	7 (7.2%)
Bronchoscopy samples	7 (7.2%)
Ascites	4 (3.4%)
Number of pathogens detected, n (%)	
One species	21 (21.9)
Two species	18 (18.6)
Three species	29 (29.9)
Species, n (%)	
*Enterococcus faecium*	17 (17.5)
*Candida albicans*	13 (13.4)
*Escherichia coli*	7 (7.2)
*Klebsiella pneumoniae*	6 (6.2)
*Enterobacter aerogenes*	6 (6.2)
*Staphylococcus epidermidis*	4 (4.1)
*Enterobacter cloacae*	4 (4.1)

Distribution of bacterial isolates in patients with sepsis-associated ACLF. Values are presented as absolute numbers and percentages. Only the most frequently isolated species are shown; additional pathogens detected in single cases, as well as less common culture media, are not displayed.

**Table 4:** Clinical characteristics of the patients with non-sepsis-associated ACLF (n = 70).

Characteristics	Study Population (n = 70)
Age, years, median [IQR]	55 [50–61.3]
Sex, n (%)	
Female	16 (22.9)
Male	54 (77.1)
BMI, median [IQR]	25.0 [23.6–29.2]
Child–Pugh classification, n (%)	
Child–Pugh Class A	2 (2.9)
Child–Pugh Class B	9 (12.9)
Child–Pugh Class C	59 (84.3)
MELD score, median [IQR]	30 [24.0–37.3]
ACLF grade, n (%)	
Grade 1	30 (42.9)
Grade 2	22 (31.4)
Grade 3	18 (25.7)
CLIF-C-ACLF score, median [IQR]	50 [43–57]
Laboratory parameters, median [IQR]	
CRP, mg/L	22.8 [9.5–47.6]
PCT, ng/mL	0.52 [0.19–1.28]
Leukocytes, /nL	8.7 [5.4–13.2]
ALT, U/L	33.4 [19.4–52.3]
AST, U/L	62 [38.4–117.8]
gGT, U/L	70 [35–143.4]
Bilirubin mg/dL	6 [2.4–24]
Quick, %	41 [30–47.3]
INR	1.7 [1.6–2.3]
Thrombocytes, /nL	63.4 [46.8–98.3]
Albumin, g/L	25.9 [20.7–32]
Organ support, n (%)	
Circulatory support (vasopressor therapy)	25 (35.7)
Respiratory support within 5 h of admission	18 (25.7)
Renal replacement therapy	2 (2.9)
Duration of ICU stay, days, median [IQR]	4.5 [2.0–7.3]
Outcome, n (%)	
Death	14 (20.0)
Discharged after subsequent transfer to general ward	47 (67.1)
Transfer to another ICU	5 (7.1)
Discharge against medical advice	2 (2.9)
Transfer to neurorehabilitation unit	2 (2.9)

Baseline demographics, disease severity, laboratory parameters, ICU course, and outcomes of patients with alcohol-related cirrhosis who developed ACLF in the absence of sepsis. Values are presented as numbers (percentages) or median with interquartile range (IQR), as appropriate.

**Table 5:** Multivariable logistic regression for in-hospital mortality in patients with alcohol-related cirrhosis and ACLF (n = 188). Results are shown as adjusted odds ratios (OR) with 95% confidence intervals (CI). An OR > 1 indicates increased odds of in-hospital mortality, whereas an OR < 1 indicates reduced odds.

Variable	*p*-Value	Adjusted Odds Ratio	95% CI for Odds Ratio
Sepsis (yes vs. no)	<0.001	4.68	2.31–9.51
Age (year)	0.796	1.01	0.97–1.04
Sex (female vs. male)	0.535	1.27	0.59–2.72
MELD score (points)	0.531	1.02	0.97–1.07
ACLF grade (2–3 vs. 1)	0.003	2.07	1.28–3.37

**Table 6:** Multivariable logistic regression for in-hospital mortality in patients with alcohol-related cirrhosis and ACLF, including SOFA Score (n = 188). Results are presented as adjusted odds ratios (OR) with 95% confidence intervals (CI).

Variable	*p*-Value	Adjusted Odds Ratio	95% CI for Odds Ratio
Sepsis (yes vs. no)	<0.001	4.88	2.37–10.03
Age (year)	0.773	1.01	0.97–1.04
Sex (female vs. male)	0.664	1.19	0.55–2.55
MELD score (points)	0.491	1.02	0.97–1.07
ACLF grade (2–3 vs. 1)	0.324	1.35	0.74–2.45
SOFA score	0.019	1.15	1.02–1.28

## Data Availability

The raw data supporting the conclusions of this article will be made available by the authors, without undue reservation.

## Abbreviations

The following abbreviations are used in this manuscript:

ACLF	Acute-on-chronic liver failure
ALT	Alanine aminotransferase
AST	Aspartate aminotransferase
BMI	Body mass index
CANONIC	Chronic Liver Failure Acute-on-Chronic Liver Failure in Cirrhosis Study
CI	Confidence interval
CLIF-C ACLF	CLIF Consortium ACLF score
CLIF-C OF	CLIF Consortium Organ Failure score
CRP	C-reactive protein
CT	Computed tomography
DILI	Drug-induced liver injury
EASL-CLIF	European Association for the Study of the Liver—Chronic Liver Failure Consortium
gGT	Gamma-glutamyl transferase
HCC	Hepatocellular carcinoma
ICU	Intensive care unit
IQR	Interquartile range
MELD	Model for End-Stage Liver Disease
NACSELD	North American Consortium for the Study of End-Stage Liver Disease
OR	Odds ratio
RRT	Renal replacement therapy
SBP	Spontaneous bacterial peritonitis
SD	Standard deviation
SOFA	Sequential Organ Failure Assessment
